# Comparative mitochondrial genomics within and among species of killifish

**DOI:** 10.1186/1471-2148-9-11

**Published:** 2009-01-13

**Authors:** Andrew Whitehead

**Affiliations:** 1 Department of Biological Sciences, Louisiana State University, Baton Rouge, Louisiana 70803, USA

## Abstract

**Background:**

This study was motivated by the observation of unusual mitochondrial haplotype distributions and associated physiological differences between populations of the killifish *Fundulus heteroclitus *distributed along the Atlantic coast of North America. A distinct "northern" haplotype is fixed in all populations north of New Jersey, and does not appear south of New Jersey except in extreme upper-estuary fresh water habitats, and northern individuals are known to be more tolerant of hyposmotic conditions than southern individuals. Complete mitochondrial genomes were sequenced from individuals from northern coastal, southern coastal, and fresh water populations (and from out-groups). Comparative genomics approaches were used to test multiple evolutionary hypotheses proposed to explain among-population genome variation including directional selection and hybridization.

**Results:**

Structure and organization of the *Fundulus *mitochondrial genome is typical of animals, yet subtle differences in substitution patterns exist among populations. No signals of directional selection or hybridization were detected. Mitochondrial genes evolve at variable rates, but all genes exhibit very low dN/dS ratios across all lineages, and the southern population harbors more synonymous polymorphism than other populations.

**Conclusion:**

Evolution of mitochondrial genomes within *Fundulus *is primarily governed by interaction between strong purifying selection and demographic influences, including larger historical population size in the south. Though directional selection and hybridization hypotheses were not supported, adaptive processes may indirectly contribute to partitioning of variation between populations.

## Background

*Fundulus heteroclitus *is a euryhaline killifish that occupies diverse habitats along steep osmotic and thermal gradients; large populations are found in extreme upper-estuary fresh water sites through brackish estuarine habitats to marine environments, and across a steep latitudinal temperature gradient along the Atlantic coast of North America from Nova Scotia (Canada) to Florida (USA). This species has long been of interest to science in part because of their extraordinary physiological resilience in the face of osmotic stress [1], and as a model for studying adaptive evolutionary variation along ecological clines [2]. To date, the bulk of evolutionary studies on this species have focused on adaptive variation related to temperature and pollution (see review in Burnett et al. [3]). However, other studies reveal unusual patterns of mitochondrial haplotype distributions that covary with differences in osmotic habitat [4] and physiological tolerance to osmotic stress [5,6]. Together, results from these studies converge on the hypothesis that natural selection has helped shape mitochondrial genomic variation as an adaptive response to physiological challenges posed by life in fresh water habitats. The purpose of this study was to compare mitochondrial genome variation within and among *Fundulus *species to test this and related hypotheses.

For coastal populations of *F. heteroclitus*, a phylogeographic break that centers on the Hudson River separates "northern" from "southern" types, with a narrow zone of introgression found in northern New Jersey. This phylogeographic break is supported by data from mitochondrial haplotype variation [4,7,8], but also from studies of variation in nuclear proteins [2,7,9], microsatellites [10,11], and gene expression [12]. Few studies in *F. heteroclitus *have examined adaptive variation associated with potential selective agents other than temperature and pollution, such as strong osmotic gradients in estuarine environments.

Hyposmotic tolerance varies widely among *Fundulus *species [13]. Though *F. heteroclitus *is widely tolerant of osmotic extremes [13], populations are distributed along steep salinity gradients in nature and within-species variation in hyposmotic stress tolerance exists. For example, adult fish from northern populations are more tolerant of hyposmotic transfer than southern populations [6], and individuals from northern populations have higher reproductive success (fertilization success and larval survival) in low salinities than those from southern populations [5]. Intriguingly, mitochondrial haplotype data show that the "northern" haplotype is never found south of the phylogeographic break, except in extreme upper estuary habitats that are exclusively fresh water [4]. In these southern fresh water habitats the northern haplotype is fixed, but only dozens of miles downstream, after transition to slightly brackish water habitats, the southern haplotype is fixed (unpublished data).

I hypothesize (as have others [4,6]) that different mitochondrial genotypes are favored by natural selection in different osmotic environments, and that the "northern" haplotype provides an adaptive advantage in hyposmotic habitats. One model that could account for the unusual contemporary distribution of mitochondrial haplotypes is as follows: As Pleistocene glaciers receded, successful invaders of newly emergent northern habitats were likely those that could tolerate low salinities leading to fixation of those genotypes during northward expansion, and those same genotypes remain fixed in southern habitats that are fresh water. It is physiologically plausible that mitochondrial genes may be involved in adaptation to fresh water habitats since hyposmotic acclimation is very energetically demanding [14,15] requiring the recruitment of mitochondria-rich cells in gill epithelia [16]. An alternate hypothesis is that mitochondrial haplotypes from the exclusively fresh water species *F. diaphanus *have introgressed into *F. heteroclitus *populations accounting for the unique haplotype distribution and hyposmotic tolerance in "northern" *F. heteroclitus*. This possibility has been recognized by others [6,17] and is plausible since *F. diaphanus *live exclusively in fresh water, co-occur with *F. heteroclitus *in fresh water and northern habitats, these two species are known to hybridize where they co-occur, and hybrids are only successful from pairings of female *F. diaphanus *with male *F. heteroclitus *[17,18]. Another alternative is that southern fresh water populations are remnants from a northward expansion of northern haplotypes following Pleistocene glaciation.

To test these hypotheses I sequenced complete mitochondrial genomes (mitogenomes) from northern, southern, and fresh water populations of *F. heteroclitus*, from *F. grandis *(the sister taxon to *F. heteroclitus*) and from *F. diaphanus*. Mitogenome sequences were used to test for the influence of neutral drift, directional selection, and selective constraint on intraspecific and interspecific variation, and to define phylogenetic relationships among populations and species.

## Methods

### Populations and species used

Complete mitochondrial genomes were sequenced from two individuals from each of five taxa including three populations of *Fundulus heteroclitus*, one population *F. grandis *(Fgds; Cocodrie, Louisiana) which is the sister species to *F. heteroclitus*, and one population of *F. diaphanus *(Fdia; Piscataway Park, Maryland) which is a co-occurring congener known to sometimes hybridize with *F. heteroclitus *[18]. *F. heteroclitus *were sampled from Maine (Fhet-ME; north of phylogeographic break, northern haplotype), Georgia (Fhet-GA; south of phylogeographic break, southern haplotype), and from an extreme upper-estuary fresh water habitat (Fhet-MDPP; south of phylogeographic break, northern haplotype). This fresh water *F. heteroclitus *population is from the Potomac River at Piscataway Park in Maryland. This is the same site as site "26" in Duvernell et al. [11] and site "PS" in Smith et al. [4], and is the same site from which *F. diaphanus *were collected for this study.

### DNA preparation, PCR, and sequencing

Genomic DNA was extracted from fresh livers using Qiagen DNeasy extraction kits, and PCR primers were from Lee et al. [19] or Miya & Nishida [20] or specifically designed for this study (see Additional file 1). Long-distance PCR (TaKaRa LA taq) was first used to amplify three overlapping regions covering the complete mitogenome sequence. Long PCR amplifications were performed in 50 μl reactions including 10 μM final concentration of each primer under cycling conditions including 40 cycles of 94°C denaturation for 30-s followed by 15-min annealing/extension at 68°C, with a final 72°C hold for 10-min. Long amplification products were used as the template for a series of 39 overlapping PCR reactions. PCR was performed in 20 μl reactions including 0.25 μM final concentration of each primer under cycling conditions including 5 cycles of 20-s denaturation at 94°C, 15-s annealing at 37°C, and 45-s extension at 72°C, followed by 25 cycles of 20-s denaturation at 94°C, 15-s annealing at 45°C, and 45-s extension at 72°C. Amplification products from both long-distance and nested PCR reactions were electrophoresed on 1% agarose to verify size and cleaned using Ampure magnetic beads (Agencourt Bioscience).

Both strands of PCR products were directly sequenced using Big-Dye Terminator cycle sequencing (Applied Biosystems) in 10 μl reactions including 0.5 μl Big Dye and 0.32 μM final concentration of sequencing primers (forward and reverse PCR primers). The cycling profile consisted of 40 cycles of 15-s denaturation at 94°C, 20-s annealing at 37°C, and 4-min extension at 60°C. Sequencing reaction products were cleaned using CleanSeq magnetic beads (Agencourt Bioscience) before electrophoresis on an Applied Biosystems 3130XL Genetic Analyzer.

### Genome investigations

A consensus sequence was determined for each individual genome following assembly of individual reads in Gap4 [21]. Complete mitogenome consensus sequences were aligned using ClustalW in MEGA 4.0 [22] and alignments annotated in SEQUIN . Transfer RNAs were identified using tRNAscan [23] and protein coding genes by alignment with *Kryptolebias marmoratus *and *Gambusia affinis *mitogenome sequences (GenBank accession numbers AF283503 and AP004422, respectively) coupled with determination of in-frame start and stop codon positions.

Genome-wide characteristics were enumerated and compared among lineages. These characteristics included genome-wide, gene-specific, and taxon-specific differences in G/C content, synonymous and amino acid replacement substitution rates, transition/transversion substitution ratios, and codon usage bias. Topological predictions of transmembrane segments of electron transport proteins were determined from the consensus prediction from multiple models [24-28] using the server-based software TOPCONS  with the sequence of individual Fhet-ME2 as the query sequence. Radical amino acid polymorphisms were identified according to the criteria outlined in Woolley et al. [29].

**Figure 1:**
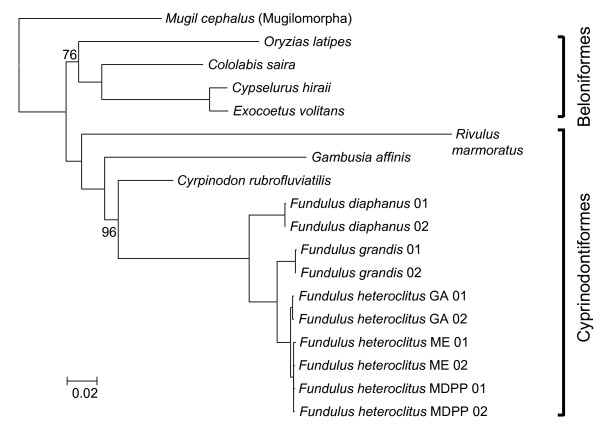
**Bayesian phylogeny of concatenated mitochondrial genome protein sequences (excluding third positions of codons) with posterior probabilities indicated only if less than 100**.

A suite of models was used to test for the influence of different evolutionary forces shaping mitogenome variation within and among species. First, dN/dS ratios were calculated for the entire mitogenome and for each gene separately to test for lineage-specific departures from neutrality (ratios significantly greater than 1.0 are evidence for directional selection). Second, the branch-sites model A test 2 [30] in CODEML (PAML 4.0, [31]) was used to test for departures from neutrality at specific substitution sites, since dN/dS analyses of whole mitogenomes and whole genes may be insensitive for detecting selection acting on a small number of important substitutions. Likelihood ratio tests were used to compare the null neutral model (model = 2, NSsites = 2, fix_omega = 1, omega = 1) against the alternate model of branch-specific positive selection (model = 2, NSsites = 2, fix_omega = 0, omega = 1.5) on the branch leading to *F. heteroclitus *northern clade. The well-supported tree indicated in Figure 1 was used as the input tree for this model. A Bayes empirical Bayes procedure [30,31] was used to test the likelihood of alternate evolutionary forces (neutral drift, purifying selection, directional selection) governing the evolution of specific substitutions along the northern branch. Third, McDonald-Kreitman tests [32] were used to test for departure from neutral evolution across *Fundulus *lineages by testing for significant excess of amino acid replacement fixations or excess of synonymous polymorphisms relative to divergence between pairs of taxa [33]. A neutrality index (NI) was calculated as the ratio of the number of synonymous fixed to synonymous polymorphic sites divided by the number of amino acid replacement fixed to replacement polymorphic sites [34], and statistical significance evaluated using the Fisher's exact test (two-tailed) performed in DnaSP [35]. Neutrality index values were calculated for pairings of *F. grandis *with each of the three *F. heteroclitus *populations. Index values significantly greater than 1.0 indicate excess amino acid polymorphism relative to divergence and significantly less than 1.0 indicate a deficiency of amino acid polymorphism [33,34,36].

Phylogenetic analyses were used to test for possible hybridization between *F. diaphanus *and co-occurring *F. heteroclitus *populations. These analyses examined the relationship of *Fundulus *populations and species (Acanthopterygii; Atherinomorpha; Cyprinodontiformes; Fundulidae) to each other and to other closely related fishes for which complete mitogenomes exist including *Cyprinodon rubrofluviatilis *(Acanthopterygii; Atherinomorpha; Cyprinodontiformes; Cyprinodontidae), *Gambusia affinis *(Acanthopterygii; Atherinomorpha; Cyprinodontiformes; Poeciliidae), *Kryptolebias marmoratus *(Acanthopterygii; Atherinomorpha; Cyprinodontiformes; Rivulidae), *Oryzias latipes *(Acanthopterygii; Atherinomorpha; Beloniformes), *Cololabis saira *(Acanthopterygii; Atherinomorpha; Beloniformes), *Cypselurus hiraii *(Acanthopterygii; Atherinomorpha; Beloniformes), *Exocoetus volitans *(Acanthopterygii; Atherinomorpha; Beloniformes), and *Mugil cephalus *(Acanthopterygii; Mugilomorpha) (GenBank accession numbers EF442803, AP004422, AF283503, AP004421, AP002932, AB182653, AP002933, and AP002930, respectively). Protein-encoding gene sequences were concatenated and aligned using ClustalW and Bayesian methods were used to reconstruct phylogenetic relationships using MrBayes 3 [37] with third codon positions excluded (Nst = 6, Rrates = Invgamma, Ngen = 10,000,000, sampling every 10 gen, nchains = 8, nruns = 2, burnin = 0.25, outgroup = *M. cephalus*, all other parameters set at default values). These analyses were run on the parallel version of MrBayes 3.1.2 compiled on the LONI high performance computing system at LSU .

## Results and discussion

### Genome-wide characteristics

*Fundulus *mitochondrial genomes (16,524–16,531 bp long; GenBank:FJ445394–FJ445403) are typical in that they contain the identical gene complement (22 transfer RNA genes, 2 ribosomal RNA genes, and 13 protein-coding genes) and gene order as found in most vertebrate mitochondrial genomes. The control region is long (865–870 bp) with many conserved blocks and found between tRNA-Pro and tRNA-Phe. The origin of light strand replication is a short highly conserved block (36 bp) found between tRNA-Asn and tRNA-Cys. Gene start sites are typically immediately downstream of the end of the previous gene, and several genes start before the end of the preceding gene. For example, the end of ATPase8 and beginning of ATPase6 overlap 10 bp, ND4L and ND4 overlap 7 bp, and the ends of ND5 and ND6 (coded on the opposite strand) overlap 4 bp. Indeed, the genomes are highly compact as only 73 bp of non-coding intergenic spacer DNA is found. All genes are encoded on the heavy chain except for ND6 and eight tRNA genes (tRNA-Gln, tRNA-Ala, tRNA-Asn, tRNA-Cys, tRNA-Tyr, tRNA-Ser (UCN), tRNA-Glu, and tRNA-Pro).

**Figure 2:**
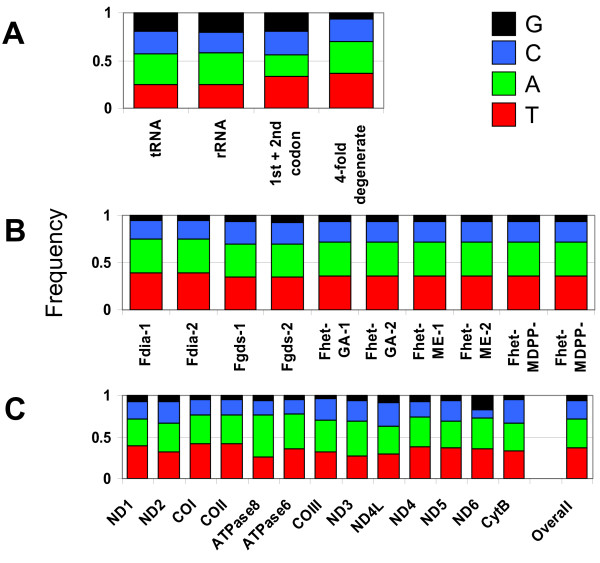
**Base composition for A) each region of the mitochondrial genome summed across all genomes sequenced, B) each protein coding region summed across all genomes sequenced, and C) for each of the 10 individual genome sequenced, including two individuals from each of *F. diaphanus *(Fdia), *F. grandis *(Fgds), *F. heteroclitus *Georgia population (Fhet-GA), *F. heteroclitus *Maine population (Fhet-ME) and *F. heteroclitus *fresh water Maryland population (Fhet-MDPP)**.

All four nucleotides are equally represented in most mitogenome regions including tRNA, rRNA, and 1^st ^and 2^nd ^positions of codons in protein coding regions (Figure 2a), though a significant bias against G-ending four-fold degenerate codon sites was detected in protein coding regions (p < 0.001, Chi-square goodness of fit test). This bias toward A-T enrichment at third position codon sites is consistent across genes except ND6 (Figure 2b), and consistent across all 10 mitogenome sequences (Figure 2c), though the bias is not nearly as dramatic as in *Drosophila *where nearly 94% of all codons end in A or T [38]. Strand-specific differences in substitution patterns are also observed. ND6 is the only gene encoded on the light chain and A→G substitutions predominate, whereas T→C substitutions predominate in the remaining genes that are encoded on the heavy chain. The same strand-biased substitution pattern is also observed in fruit flies [39] and these genome-scale phenomena may be the result of interaction between mutational tendencies and selection for translation efficiency [33].

**Figure 3:**
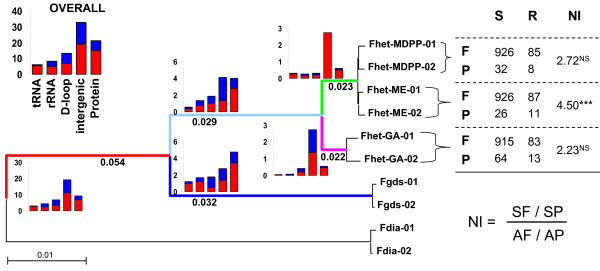
**Overall and branch-specific relative transition (red) and transversion (blue) mutation rates (substitutions per 100 bases) for different genomic regions, and branch-specific dN/dS ratios (number below branch)**. Table includes number of pairwise (with *F. grandis*) synonymous (S) or amino-acid replacing (R) and fixed (F) or polymorphic (P) mutations, including neutrality index (NI) from McDonald Kreitman (MK) test performed in dnaSP. Significance of MK is tested is indicated as follows; * 0.01 < P < 0.05; ** 0.001 < P < 0.01; *** P < 0.001 for Fisher's exact test with Bonferroni correction for multiple tests. Colored lines indicate lineages referred to in Figure 5.

Mutation rates vary across genomic regions, but only subtle differences appear among lineages. Non-coding intergenic spacer regions have the highest mutation rate (33% of sites were variable among all 10 *Fundulus *genomes) followed by protein coding regions (21% of sites variable) (Figure 3). The lowest mutation rates are in tRNA (6% of sites variable) followed by rRNA (8% of sites variable) and the D-loop (13% of sites variable). This pattern appears inconsistent across lineages. In branches separating sister taxa *F. heteroclitus *and *F. grandis*, rates of substitution in non-coding intergenic spacer regions appear to decrease to match or fall behind substitution rates in protein coding regions (Figure 3). However, only 73 bases constitute intergenic non-coding DNA, so such a small proportion of the overall genome may be subject to spurious differences in mutation rate across lineages given the small number of genomes sequenced. Similarly, transition/transversion rate differences in intergenic regions are likely to be spurious; that is, apparent intergenic transition/transversion differences between *F. heteroclitus *lineages are due to just one mutation. Excluding intergenic DNA, few differences remain among lineages in relative mutation rates of tRNA, rRNA, D-loop, and protein coding regions, except that in the *F. heteroclitus *Georgia lineage tRNA and rRNA mutation rates are disproportionately lower and D-loop mutations disproportionately higher than the overall pattern (p < 0.001, Chi-square goodness of fit test).

### Evolutionary mechanisms

**Figure 4:**
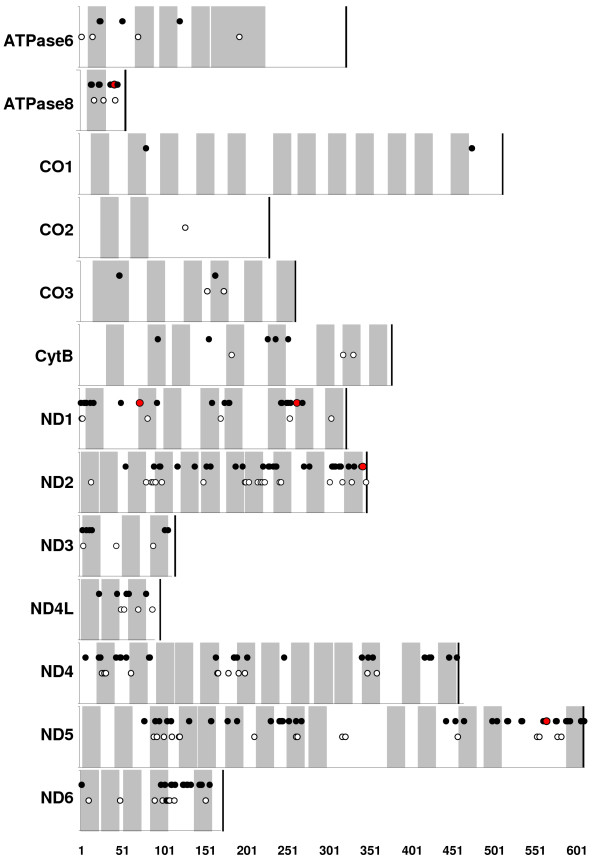
**Topological predictions of transmembrane domains for mitochondrially-encoded electron transport proteins and location of amino acid polymorphisms in *Fundulus***. The X-axis represent amino acid site for each protein, and predicted transmembrane regions are highlighted by gray bars, where white bars represent loop regions. Positions of amino acid sites that are polymorphic among the 10 mitochondrial genomes sequenced are indicated by circles. Radical amino acid polymorphisms are highlighted as closed circles, and polymorphisms that are fixed exclusively in the northern *F. heteroclitus *lineage are highlighted in red.

Amino acid polymorphisms tend to be unevenly distributed across proteins (Figure 4); regions of sparse amino acid polymorphism, especially of sparse radical polymorphism, could be the result of stronger purifying selection due to the presence of functional domains. Indeed, transmembrane regions of ND2, ND4, and ND5 are located in the membrane-embedded arm of the NADH dehydrogenase complex and likely play an active role in proton pumping [40], and in *Fundulus *these regions harbor significantly fewer radical amino acid polymorphisms (p > 0.05, Chi-square test of independence) relative to the loop regions of these proteins (Figure 4) which is consistent with data from mammals [41].

Only five amino-acid replacement mutations are fixed exclusively in the northern lineage (including Maryland fresh water and Maine individuals: green lineage in Figure 3). Two occur in ND1 (Pro→Ser at amino acid site 73, Thr→Ala at site 264) and one in each of ND2 (Ala→Thr at site 344), ATPase8 (Glu→Asp at site 42), and ND5 (Ile→Val at site 555). All ND subunits are located in the membrane-bound portion of the NADH dehydrogenase complex; ND1 is thought to be located at the junction between the matrix arm and membrane-embedded arm, and ND2 and ND5 are located in the membrane-embedded arm where proton pumping occurs [40]. ATPase subunit 8 is a core component of the F0 complex of ATP synthase and may serve an important role in subunit assembly [42]. Though these substitutions could represent candidate loci for directional selection, statistical support is not strong. Branch-site tests indicate low posterior probabilities (less than 50%) for a model of positive selection in the northern lineage for each of these five sites. However, given the relatively limited phylogenetic sampling included in this study (only five taxa) the branch-sites tests may have insufficient power to detect positive selection [43], and more thorough phylogenetic sampling within *Fundulus *may increase statistical support for positive selection at these candidate loci. Only one amino acid replacement is shared between *F. diaphanus *and the northern populations of *F. heteroclitus *(Ile→Val at site 592 at edge of inner membrane region in ND5), offering little support of convergent mutation conferring hyposmotic tolerance between these fresh water tolerant and co-occurring groups. Over 160 synonymous mutations distinguish northern from southern populations of *F. heteroclitus*, though no lineage-specific codon usage biases were evident.

**Figure 5:**
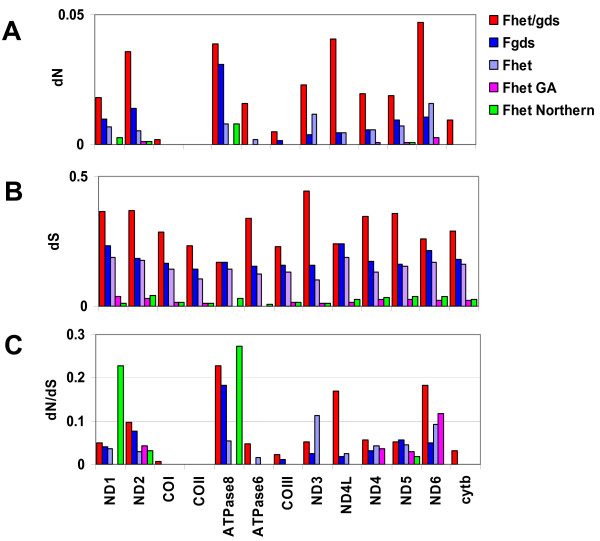
**Number of non-synonymous (A) and synonymous (B) mutations per site, and dN/dS ratio (C), for each gene and for each lineage**. Colors indicate lineage, and match those in Figure 3. Note that Y-axis scales are different for each sub-figure.

**Figure 6:**
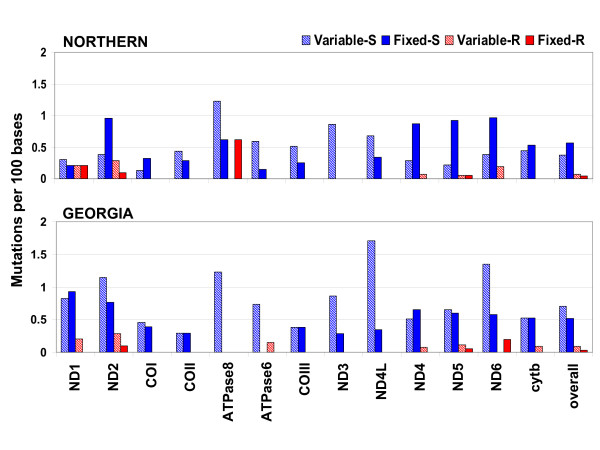
**Mitochondrial protein sequence variation within and among northern (Maine and Maryland fresh-water) and Georgia populations**. Blue hatch bars represent synonymous mutations that are variable within population (Variable-S), blue solid bars represent synonymous mutations that are fixed between populations (Fixed-S), red hatch bars represent amino acid replacement mutations that are variable within population (Variable-R), and red solid bars represent amino acid replacement mutations that are fixed between populations (Fixed-R).

In protein coding regions, mutation rates indicate that mitogenome proteins evolve at variable rates, and patterns of substitution differ between populations of *F. heteroclitus*. Ratios of synonymous to non-synonymous mutations indicate that NADH dehydrogenase (ND) genes are evolving relatively quickly whereas cytochrome oxidase (CO) genes are evolving relatively slowly (Figures 5 and 6), consistent with findings in mammals [44]. Indeed, branch-site models indicate that more sites are evolving according to a model of neutral evolution (in contrast to a model of strong selective constraint) in the ND genes than in the CO genes (on average 1.79 sites per 100 for ND genes compared to 0.14 sites per 100 for CO genes). Very low dN/dS ratios are characteristic for all genes across all lineages (Figures 3 and 5), consistent with strong selective constraint governing mitogenome evolution. However, subtle differences appear in patterns of amino acid variation within and between Georgia and Maine populations, as reflected by differences in the neutrality index.

Comparisons of fixed and variable synonymous and replacement mutations between each *F. heteroclitus *population and *F. grandis *allow calculation of the neutrality index (NI) which is the ratio of synonymous fixed sites to synonymous polymorphic sites divided by the ratio of replacement fixed sites to replacement polymorphic sites (Figure 3) [34]. Note that only two genomes were sequenced per taxon, so rates of within-group polymorphism are certainly underestimated but should not be biased among taxa being compared. NI values greater than 1.0 are common for animal mitochondrial genes [33,45], where amino acid variation is higher within taxa than expected relative to variation between taxa. This observation rejects neutral expectations, and excess amino acid polymorphism may reflect either the influence of balancing selection or selection against slightly deleterious mutation. All *F. heteroclitus *populations show a general pattern of NI values significantly greater than 1.0, though this pattern is most striking in the Maine population which is the only population in which the NI remains significantly greater than 1.0 after Bonferroni adjustment of p-values. This observation is consistent with the slightly deleterious model since historical population sizes are considered much larger in Georgia compared to northern populations [9,11], and excess deleterious mutation should be harder to detect in larger populations because of the proportionately greater efficiency of purifying selection. These results are consistent with NI values from *Drosophila *taxa of different historical population sizes [33], but do not reject the alternative explanation that balancing selection may be maintaining functional polymorphism in the Maine population.

Georgia individuals harbor twice the number of synonymous mutations compared to northern populations (Figures 3 and 6), again likely reflecting much larger historical population size in which synonymous mutations are more likely to be maintained but mildly deleterious replacement mutations are efficiently purged. Indeed, synonymous polymorphic sites are consistently fewer across genes within the Maine population compared to the Georgia population (Figure 6), and chi-squared tests of 2 × 4 contingency tables indicate that the Georgia mutation ratios are significantly different from those of both northern populations (p > 0.01 for both comparisons), but northern ratios are not different from each other (p = 0.77).

In agreement with very low dN/dS ratios, branch-site tests indicate that over 95% of substitutions appear to be evolving according to a model of strong selective constraint (substitutions with posterior probabilities > 90% for site class 0 using BEB in CODEML) across lineages. In the lineage leading to *F. heteroclitus *northern clade only 33 sites (out of 3,813 possible sites) reject the evolutionary model of strong selective constraint, and all 33 of these sites best fit a model of neutral evolution. Similarly, the vast majority of substitutions along all other branches in the phylogeny best fit a model of strong selective constraint.

Polymorphism in transcriptional control regions may affect protein expression levels, and therefore be phenotypically important. Presumed functional domains within the teleost control region, such as conserved sequence blocks (CSB) in the central conserved region, CSB-II, and CSB-III (as identified in Lee et al. [46]), are all highly conserved across *Fundulus *species. The only mutation unique to northern populations within these regions is at the beginning of the central conserved region (C→T at mitogenome position 15958 in *F. heteroclitus *ME-2), but the functional importance of this polymorphism has yet to be determined.

Together, these lineage-specific patterns of substitution, dN/dS ratios, and branch-site tests offer little support for the influence of direct selection on mitogenome evolution within *Fundulus heteroclitus*. Though five amino acid substitutions are unique to the northern clade, lineage-specific mitogenome differences more likely reflect differences in historical population size. Low dN/dS ratios coupled with results from branch-site tests indicate the influence of strong selective constraint, and an excess of synonymous polymorphisms within the Georgia population (relative to northern populations and relative to replacement polymorphisms within Georgia) is consistent with larger historical population size. Nuclear data also indicate greater genetic diversity in southern versus northern populations of *F. heteroclitus *[9,11]. Larger populations can harbor more purely neutral mutations and are more efficient at purging slightly deleterious mutations [47,48]. These results support the model that mitogenome evolution in killifish is primarily governed by interaction between strong selective constraint and demographic parameters, which is consistent with broader patterns of mitogenome evolution in other organisms including flies [49], copepods [50], rats [44], and humans [51].

### Phylogenetic analyses

Phylogenetic analyses of concatenated mitogenome protein sequences indicate that within *Fundulus *the Maine population and the Maryland fresh water population mitogenome types are very similar, all three *F. heteroclitus *populations are monophyletic and form a clade with sister species *F. grandis*, and *F. diaphanus *is the outgroup (Figure 1). Posterior probabilities for branching nodes were high, and sequence from two nuclear genes supports the same topology (data not shown). Broader analysis of all complete mitochondrial genomes (concatenated proteins) that are currently available for the clade indicate that cyprinodontiformes and beloniformes fishes are reciprocally monophyletic (Figure 1), in contrast to the findings reported in Miya et al. [52]. Furthermore, *Fundulus *taxa appear to be relatively recently derived among the cyprinodontiformes lineages. These data provide no evidence for hybridization between fresh water *F. heteroclitus *and *F. diaphanus *in the upper Chesapeake estuary, and thus does not contribute to explaining the unusual distribution of mitochondrial genotypes in extreme upper-estuary habitats.

## Conclusion

These comparative data illuminate the relative influence of evolutionary forces that shape mitochondrial genome variation in killifish, but do not offer strong support for hypotheses proposed to explain unusual distributions of mitochondrial types in nature. Patterns of synonymous and amino acid replacement substitutions and branch-site models of codon substitution provide little evidence that directional selection has directly influenced mitochondrial genome sequence variation between southern, northern, and fresh water populations of *F. heteroclitus*. However, five amino acid replacements and one mutation in a conserved and presumably functional block of the control region are unique to the northern clade and may be candidates for further study. Also, since all *F. heteroclitus *mitogenomes form a monophyletic group with *F. diaphanus *as the out-group, there is no evidence for introgression with *F. diaphanus *in northern and fresh water sites. Substitution patterns indicate the important role of purifying selection governing mitochondrial genome evolution, and the most apparent lineage-specific patterns of variation within *F. heteroclitus *is an excess of synonymous relative to replacement polymorphism in the Georgia population (Figure 3). This could reflect a larger historical population size, in which synonymous (purely neutral) polymorphisms are retained but slightly deleterious replacement variants are purged.

Since these data do not support two explicit hypotheses, the evolutionary mechanisms responsible for maintaining "northern" mitochondrial types in southern fresh water habitats remains unresolved. One possible explanation is that fresh water types are relicts from an historical northward expansion following Pleistocene glaciation. An alternate explanation is that southern types that reside in brackish habitats are competitively excluded from contributing to the gene pool in nearby fresh water habitats where northern types reside. We know that transition from fresh water to only slightly brackish demands considerable physiological adjustment [53]. We also know that northern types have higher fitness [5] and better osmotic compensatory ability [6] than southern types in fresh water. One hypothetical mechanism keeping gene pools distinct may be through breakdown of intergenomic coadaptation where unmatched nuclear-mitochondrial hybrids exhibit lower fitness (reviewed in Rand et al. [54]). One way to test the competitive exclusion and relict population hypotheses would be to densely sample populations along the salinity cline to test for sharp inflections (indicating competitive exclusion) or gradual transitions (indicating introgression) in allele frequencies across the ecological boundary between fresh water and slightly brackish habitats. This work is ongoing in the Whitehead laboratory.

## Authors' contributions

AW designed experiments, carried out laboratory work and statistical analysis, and drafted the manuscript.

## Supplementary Material

Additional file 1 **Table 1: Primers used for PCR and sequencing of *Fundulus *mitochondrial genomes.** A list of primer names and sequences used for PCR and sequencing. Click here for file
